# The long-term effects of adolescent pregnancies in a community in Northern Ghana on subsequent pregnancies and births of the young mothers

**DOI:** 10.1186/s12978-017-0443-x

**Published:** 2017-12-29

**Authors:** Anne-Sophie Yussif, Anyetei Lassey, Gabriel Yao-kumah Ganyaglo, Eva J. Kantelhardt, Heike Kielstein

**Affiliations:** 1 Department of Anatomy and Cell Biology, Martin Luther University Halle-Wittenberg, Faculty of Medicine, Halle (Saale), Germany; 2 Department of Obstetrics and Gynaecology, University of Ghana Medical School, Accra, Ghana; 3 Korle Bu Teaching Hospital, Guggisberg Avenue, Accra, Ghana; 4 Faculty of Medicine, Department of Gynecology, Martin Luther University Halle-Wittenberg, Halle (Saale), Germany

**Keywords:** Adolescent pregnancy, Ghana, Caesarean section, Stillbirth

## Abstract

**Background:**

In Ghana, adolescents represent 22% of the total population. The rates of adolescent pregnancies are high. Of all births registered in the country in 2014, 30% were by adolescents, and 14% of adolescents aged between 15 and 19 years had begun childbearing. Pregnancies and deliveries of adolescents are accompanied by more risks as compared to older women. The aim of the study was to explore the long-term effects of adolescent pregnancies on subsequent pregnancies and births and on the socioeconomic status of the women.

**Method:**

A cross-sectional interviewer-performed survey of a purposive sample of 400 women in one community of Northern Ghana was conducted. Relationships between the age at first pregnancy and complications such as cesarean section, preterm or stillbirth and others were explored in 143 patients using the statistical program SPSS (Statistical Package for the Social Sciences).

**Result:**

Results show that adolescent women (<19 years at their first pregnancy) have an 80% higher risk for a cesarean section for the first and subsequent births as compared to older women (≥ 19 years). Furthermore, younger mothers have a 45% higher risk of stillbirths and a 30% increased risk of losing their baby within the first 6 weeks after birth. There was no difference in the socioeconomic status between the two age groups.

**Conclusion:**

Adolescent pregnancies are risk factors for the outcome of subsequent pregnancies of these mothers. This study, for the first time, shows that not only the first pregnancy and birth of very young women are negatively influenced by the early pregnancy but also subsequent pregnancies and births. While this study is of a purposive sample of women in one community, the clinical relevance of this study should not only be interesting for healthcare practitioners in Northern Ghana and other African regions but also for prevention campaigns in these regions.

## Plain English summary

Worldwide, the rates of adolescent pregnancies are high. Pregnancies and deliveries of young women (< 19 years at their first pregnancy) are accompanied by more risks as compared to older women. The aim of the study was to investigate the long-term effects of adolescent pregnancies on subsequent pregnancies and births. Furthermore, the consequences on the socioeconomic status of the women was evaluated.

We interviewed a purposive sample of 143 women in Northern Ghana about their pregnancies and births. Thereafter, relationships between the age at first pregnancy and birth complications of the first and all following births were explored.

In this community sample results show that adolescent women have a higher risk for a cesarean section for the first and following births as compared to older women. Furthermore, younger mothers have a higher risk of stillbirths and of losing their newborn baby. There was no difference in the socioeconomic status between the two age groups.

This study, for the first time, shows that not only the first pregnancy and birth of very young women are negatively influenced by the early pregnancy but also following pregnancies and births.

## Background

The Ghanaian healthcare system is divided into two sectors, namely the formal sector and the informal sector (which is also referred to as the “traditional” healthcare sector). In 2012, the physician-to-population ratio in Ghana was 1: 10,452 while the nurse-to-population ratio was 1: 1, 251 [1]. In comparison to other countries of Sub-Saharan Africa, Ghana made some progress towards the attainment of Millennium Development Goals (MDG) 4 which was focused on reducing under-five mortality (i.e. it fell by 46% instead of the 66% targeted) and the MDG 5 which was focused on reducing maternal mortality (recorded a fall of 40% instead of the targeted 75%, [2, 3].

The Northern Region is the largest region in the country with Tamale as its capital town and the second largest city by area size in Ghana. Among the indigenous communities in the Tamale Metropolis is the suburb called Yong Dakpem Yili.

In Ghana, adolescents represent 22.4% of the total population. The rates of teenage pregnancies are high. Thirty percent (30%) of all births registered in Ghana in 2014, were by adolescents, and 14% of adolescents aged between 15 and 19 years had begun childbearing [4]. The World Health Organization (WHO) fact sheet from 2015 [5] states that around 16 million adolescents give birth each year. Pregnancies and deliveries of adolescents (10–19 years old) are accompanied by more risks as compared to older women [4, 5]. Stillbirths and losing a baby within 6 weeks after the birth of children from adolescent mothers are up to 50% higher as compared to the children of mothers between the age of 20 and 29. [5] Furthermore, children of young mothers are more likely to be preterm, whereas the delivery can be prolonged. Additionally, the birth weight may also be decreased [5]. The higher risks of stillbirth in adolescent pregnancies need to be discussed as to whether the risks for young mothers are higher because they are young or because it is their first pregnancy, or both. Waldenstrom et al. found that the risk of stillbirth is higher in nulliparous women and it increases with advanced maternal age [6]. The WHO records show that adolescent pregnancies are a worldwide issue, both in industrialized and developing countries. This is reflected by various studies from different countries [7–10]. Few studies from Ghana exist, describing adolescent pregnancies [7, 11, 12]. To date, all published studies analyze the effect of adolescent pregnancies on that particular early pregnancy/birth.

The aim of the present study is to evaluate the long-term adverse consequences of adolescent pregnancies on the subsequent pregnancies and births of these young mothers. It furthermore aims to address the question of whether the socioeconomic status of the adolescent mothers was significantly affected by the early pregnancy compared to older mothers.

## Methods

### Participants

Women of different age groups in and around Yong Dakpem Yili, who could not afford to visit a hospital, were offered a free consultation at an outreach clinic from April 23rd 2012 until April 26th 2012 in Tamale. Thus, only women with actual health complaints were considered in the study. There was no age restriction for participation in the study. Four hundred (400) women with the help of nurses from the ‘Community Nurses Health Training School’ completed the questionnaires for the present study. The nurses were taught how to fill out the questionnaires several days before the start of the study. Additionally, the nurses had to translate the questionnaire in local languages / dialects since many of the women could not speak English. The study was conducted in accordance with the Helsinki Declaration of 1975 as revised in 2013***.*** The principle of informed consent was fully respected. The right of refusal to participate or withdrawal from the study at any time without undesirable consequences was confirmed to the women each time.

### Questionnaire

The questionnaire consisted of 90 questions which were divided into two categories; “Socio-demographic questions” and “Medical record”. The second category was divided further into “Present” and “General” health problems.

The socio-demographic questions included age, place of birth, marital status (single, married, divorced, widowed), year of marriage, ethnic group (Akan, Dagombe, Ewe, Ga, others), religion (Christian, Moslem, Animist, Atheist), place of domicile (rural, urban slum, urban), highest educational achievement (Tertiary, Senior High School, Junior High School, Primary, no formal education), employment (unskilled, semi-skilled, skilled, unemployed, in school) and profession.

Present medical records included the present complaints which brought the patient to the clinic, acute or chronic ailment, and frequencies of general physician and gynecologist consultations.

The category ‘General medical records’ summed up questions about the women’s menstrual period (age at first menses, frequency, length, pains), contraceptive use, number of children, pregnancies (when was the first pregnancy, how many), abortions, stillbirths, premature births, cesarean sections, perineal rupture, losing a child within the first 6 weeks after birth and other birth complications.

### Statistics

After encoding numbers whenever necessary, data was analyzed with the program SPSS. The influence of adolescent pregnancy versus no adolescent pregnancy on outcome related factors of all pregnancies was analyzed. In order to evaluate statistically significant results, the Pearson’s Chi-Square-Test and the Fisher’s exact Test were used. Odds-Ratio, *p*-value and 95% confidence interval were also calculated by SPSS. The figures depict mean values.

## Results

### Socio-demographic characteristics

Women did not answer to all the questions in each questionnaire. Therefore, the number of responses of the participants did not add up to 400 for each question. The ages of all women ranged from 13 to 80 years. Fifty percent (50%) of the women were aged 35 years or younger. The mean value was 37.8 (± 14.6 Standard Deviation, SD) years. The majority (331) of the 400 women were married, 35 widowed and 19 were single at the time; 3 of them were in a stable relationship while four were divorced. About 75% of the participants married at 20 years or earlier. The responses ranged from 10 to 40 years. The mean age at marriage was 19 (± 4.7 SD) years. The Dagombas were the largest ethnic group accounting for 365 of the participants. There were almost five times as many Muslims (*n* = 326) as Christians (*n* = 68) among the participants. There were no atheists or women who practiced African traditional religion. About 95% of the women had no formal education; 9 completed Primary School and only 6 had completed Junior High School and 3 Senior High School. Most women engaged in petty trading.

### Age groups

The study could be performed with a cohort of 143 women, since these women were certain of their age at first pregnancy (Table 1). The age at first pregnancy ranged from 10 to 40 years. About 50% of the women were younger than 20 years and 25% were 25 years or older. The mean age was 21 (± 6 SD) years. The 143 participants were divided into two groups: namely, “Age group 1” (i.e. <19 years at first pregnancy; *n* = 46) and “Age group 2” (i.e. ≥19 years at first pregnancy; *n* = 97). There was no significant difference between them with respect to the mean number of children (3.7 vs. 4.1) and the range of children (1–8 vs. 1–11).

**Table 1:** Comparison of the two age groups

	Age group 1 (n = 46)	Age group 2 (n = 97)
Age		
Min	18	20
Max	70	80
Mean (± SD)	33.3 (± 11.7)	40.6 (± 13.3)
Marital status		
Single	0	0
In a relationship	0	1
Married	41	92
Divorced	0	0
Widowed	3	4
Age at marriage		
Min	10	10
Max	35	40
Mean (± SD)	17.3 (± 5.2)	22.2 (± 6.1)
Ethnic group		
Dagombe	41	92
Gonja	1	1
Frafra	0	1
Falani	1	0
Religion		
Christian	8	18
Moslem	36	80
Animist	0	0
Atheist	0	0
Highest educational achievement		
Tertiary	0	0
Senior High School	0	0
Junior High School	0	0
Primary	0	2
No formal education	40	74
Age at 1st pregnancy		
Min	10	19
Max	18	40
Mean value (± SD)	14.8 (± 2.4)	24.1 (± 5.0)
Mean Number of pregnancies	4.6	5.0
Mean Number of children	3.7	4.1

Age group 1: women with first pregnancy <19 years (n = 46); Age group 2: women with first pregnancy ≥19 years (n = 97); SD = Standard deviation

### Abortion

**Fig. 1:**
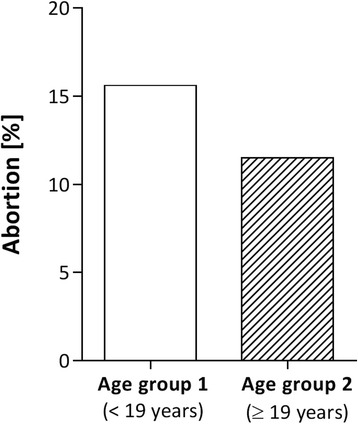
Percentage of lifetime abortions in both age groups. Age groups are defined as age at first pregnancy. They are depicted on the X-axis. The percentages are shown on the Y-axis

About 15 % (15.6%) in Group 1 and 11 % (11.5%) in Group 2 had had an abortion in the past (Fig. 1). The odds-ratio for women aged <19 years at their first pregnancy was 1.37 compared to women in age group 2 (Confidence Interval, CI_95%_ = (0.49; 3.80)). Thus, the younger women had a 37% higher abortion rate as compared to the older pregnant women, though the finding is not significant.

### Cesarean sections

**Fig. 2:**
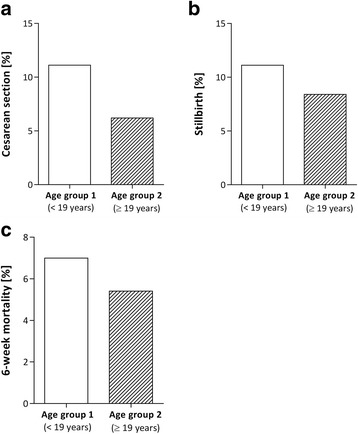
Percentage of lifetime events of **a**) cesarean sections, **b**) stillbirth, and **c**) losing a baby within the first 6 weeks after birth in both age groups are depicted on the X-axis; the percentages are shown on the Y-axis

Overall, 7.8% of the women enrolled in the present study had had a cesarean section in the past (Fig. 2a). In Group 1 five women (11.1%) had had a cesarean section while Group 2 there were six women (6.3%). The odds-ratio for cesarean section of women aged <19 years at their first pregnancy was 1.80 (CI_95%_ = (0.50; 6.50)). Thus, very young mothers have an 80% higher risk for a cesarean section as compared to older mothers. This is not only related to the first delivery when the mothers are <19 years old but also to the subsequent deliveries.

### Stillbirth and loss of child after birth

Overall 7.1% of the women enrolled in the study had had one or two stillbirths in the past (Fig. 2b). In Group 1, 8.9% of the women had had a stillbirth compared to 6.3% in Group 2. The odds-ratio for women aged <19 years at their first pregnancy was 1.44 (CI_95%_ = (0.38; 5.40)). In Group 1, 7% of the women lost their baby within 6 weeks after birth. The corresponding percentage for Group 2 is 5.4%. (Fig. 2c). The odds-ratio for women aged < 19 years at their first pregnancy was 1.30 (CI_95%_ = (0.30; 5.70)). Thus, the risk for a stillbirth in Group 1 is increased by 44% while the risk of losing a baby within 6 weeks after birth compared to 30% in older mothers. This is not only related to the first delivery when the mothers were <19 years old but also to the subsequent deliveries.

### Other complications

No further differences in terms of other birth complications, such as pain of labor, umbilical cord prolapse or strong bleedings, could be detected between the age groups. The participants were also asked if they were suffering of FGM (Female Genital Mutilation). None of the women stated to be mutilated.

### No impairment of socioeconomic factors

No significant differences could be found between the two age groups concerning socioeconomic factors. The marital status was composed as follows: 93% married in age group 1 and 95% in group 2, 7% widowed in group 1 and 4% in group 2. The mean marital age was 19 (± 4.7 SD) years, with a range from 10 (13 women) to 40 years. There was no difference in the relationship status between the two age groups. School education, formation and profession were comparable in both age groups. Only 2.7% of women of age group 2 (women ≥ 19 years) stated to have finished Primary School. No woman finished Primary School in age group 1 (women <19 years at first pregnancy). In both groups no woman visited Junior or Senior High School nor attended a tertiary institution. More than 90% of women of both age groups earn their livelihood with trading.

## Discussion

Adolescent women become pregnant in all continents, and in both the developing and the industrialized countries. Several studies describe these early pregnancies / deliveries in different countries and conclude rather contradictory consequences for the adolescent mothers and their babies [8–10, 13, 14]. The aim of the present study was the evaluation of long-term consequences of adolescent pregnancies in in a purposive sample of women in one community of Northern Ghana. Thus, impairments of all pregnancies and deliveries from women who had an adolescent pregnancy were compared with all pregnancies from mothers without adolescent pregnancy. The majority of the women enrolled in this study never had an abortion (87.2%). These results are similar to other studies [15–17]. Interestingly, the present evaluation could show that women <19 years at their first pregnancy have a 37% higher risk to have an abortion in their lifetime compared to older mothers. In a recent cross-sectional study, Adjei et al. showed comparable results [18]. The reported case of abortions (induced and spontaneous) in a total of more than 3000 women was 13.6%. Interestingly, pregnant women aged 20–29 years were 43% less likely to have an abortion as compared to younger women within the ages of 13–19 years. One possible explanation for this finding is that adolescent women are under strict parental (or guardian) control. They fear the rejection of the parents which will lead to unsafe abortions [18]. A study performed in Burkina Faso reported that women under parental control were seven-fold as likely to have induced abortion as compared ‚un-controlled’ women [19]. Another explanation, demonstrated by the Ghana Maternal Health Survey, could be the lack of money and herewith the fear to be unable to cater for the newborn baby [20]. This may result in the use of abortions as a family planning option for young women. Other reasons could also lead to spontaneous abortions in adolescent pregnancies. Lack of a good nutritional status [21] and lack of partner support [22] may lead to spontaneous abortions. This urgently demands the need for awareness campaigns and support for contraception.

Another result of the present study was the link between the age at first pregnancy and the overall cesarean section rate. The risk for a cesarean section was 80% higher for adolescents as compared to older mothers. Various reasons exist for the conduction of a cesarean section, e.g. acute fetal distress or a mismatch between the proportions between female pelvis and fetus [23]. Pregnant adolescents have different risks during pregnancy and delivery. However, more studies exist showing decreased risk for cesarean sections for younger pregnant women as compared to older women [13, 24]. Al-Haddabi et al. showed that a decreased cesarean section rate in young mothers was attributed to the fact that older women delivered heavier babies as compared to the adolescent women [13]. On the other side a study from Southern India comes to the same result as our investigation. They demonstrate a significantly higher cesarean section rate in adolescent mothers as compared to older women. Underlying reasons were predominantly fetal distress [25].

Our study shows that the risk to have a stillbirth in lifetime is more than 40% higher in adolescent mothers as compared to older mothers. Various reasons exist for stillbirth or child mortality during delivery. Several studies state, that malaria, helminthic infections, severe birth trauma and different maternal diseases, such as pre-eclampsia lead to stillbirths [26, 27]. A Nigerian study shows, that the stillbirth rate is higher in unbooked pregnant women coming to the hospital. Adolescent pregnant women are also more likely to have no prior antenatal care [28]. Furthermore, socioeconomic deprivation may end up in stillbirth [29].

Lastly, the influence of adolescent pregnancies on the socioeconomic status of the young mothers later on was evaluated. No significant influence could be detected. However, it has to be taken into consideration that all women enrolled in the present study had a low socioeconomic status, with 95% of women without completing the Primary School. A study from Althabe et al. [30] investigating 269,273 women in six low-middle income countries showed that pregnancy among adolescents is associated with impaired perinatal outcomes, being more likely associated with biological immaturity, than with socio-economic factors or inadequate delivery care. Conde-Agudelo et al. [31] performed an investigation with 854,377 Latin American women and could demonstrate that adolescent pregnancy is independently associated with increased risks of adverse pregnancy outcomes, such as low birth weight and high early neonatal mortality.

Studies performed in South Africa showed positive associations between decreased numbers of adolescent pregnancies and school enrollment or between the knowledge of contraceptive methods and a higher socioeconomic status [32, 33]. Perhaps, it is difficult to compare circumstances of life in two spatially and culturally distinct countries in Africa. Enormous efforts have been made successfully by the MDG 4 and 5 to reduce maternal and child health inequalities [34]. Other campaigns aimed to reduce the socioeconomic inequalities in Ghana and in other countries all over the world. However, women in rural Northern Ghana still have serious financial difficulties and are almost excluded from educational programs. Therefore, the sorrowful implication of this result is not that an adolescent pregnancy impairs the socioeconomic status of the young woman but that the socioeconomic status of women in a purposive sample of women in one community of Northern Ghana in general is alarming low.

The limitations of our study are the retrospective nature of the questionnaire. This may lead to inaccuracy especially concerning complications in pregnancy. A longitudinal study with a higher number in more than one community of participants was not possible due to limited financial and timely resources. Secondly, it is known that women often do not know about early abortions and also forget about abortions. This may lead to an underestimation of abortion - but probably similar in our comparison of age group 1 and 2. Thirdly, the social status was very homogeneous among all participants, the questions were not suitable to detect differences as result of an effect of age at first pregnancy.

## Conclusions

The findings of this study have shown that women with adolescent pregnancies experience more abortions, cesarean sections and stillbirths in their lifetime. All enrolled women (adolescent and older mothers) had a worrisome low socioeconomic status. These findings urgently highlight the need for extra efforts to ensure that health promotion campaigns, family planning services and educational programs reach women in Northern Ghana.

## Abbreviations

CI: Confidence interval
MDG: Millennium development goals
SD: Standard deviation
SPSS: Statistical package for the social sciences
WHO: World Health Organization

## References

[1] Peace FM, Ghana. 2014. Ghana's doctor-to-patient-ratio worsens. http://www.peacefmonline.com/pages/local/health/201406/205105.php Accessed 27 Nov 2017.

[2] Singh K, Osei-Akoto I, Otchere F, Sodzi-Tettey S, Barrington C, Huang C (2015). Ghana’s National Health insurance scheme and maternal and child health: a mixed methods study. BMC Health Serv Res.

[3] Osei-Assibey E, 2010 Population & Housing Census Report. Millennium development goals in Ghana. Ghana statistical service. 2013. http://www.statsghana.gov.gh/docfiles/2010phc/MDG%20report%20%2824-10-13%29.pdf. Accessed 1 Jan 2017.

[4] Graphic online, Accra, Ghana. 2016. Teenage pregnancy in Ghana: assessing situation and moving forward. https://www.graphic.com.gh/news/general-news/teenage-pregnancy-in-ghana-assessing-situation-and-moving-forward.html. Accessed 27 Nov 2017.

[5] World Health Organization. Adolescent pregnancy. 2016. http://www.who.int/mediacentre/factsheets/fs364/en. Accessed 01 January 2017.

[6] Waldenstrom U (2015). Advanced maternal age and stillbirth risk in nulliparous and parous women. Obstet Gynecol.

[7] Gyesaw NYK, Ankomah A (2013). Experiences of pregnancy and motherhood among teenage mothers in a suburb of Accra, Ghana: a qualitative study. Int J Womens Health.

[8] Ayuba II, Gani O (2012). Outcome of teenage pregnancy in the Niger delta of Nigeria. Ethiop J Health Sci.

[9] Lao TT, Ho LF (1997). The obstetric implications of teenage pregnancy. Hum Reprod.

[10] Shrim A, Ates S, Mallozzi A, Brown R, Ponette V, Levin I (2011). Is young maternal age really a risk factor for adverse pregnancy outcome in a Canadian tertiary referral hospital?. J Pediatr Adolesc Gynecol.

[11] Orish VN, Onyeabor OS, Boampong JN, Aforakwah R, Nwaefuna E, Iriemenam NC (2012). Adolescent pregnancy and the risk of plasmodium falciparum malaria and anaemia-a pilot study from Sekondi-Takoradi metropolis, Ghana. Acta Trop.

[12] Morhe ESK, Tagbor HK, Ankobea FK, Danso KA (2012). Reproductive experiences of teenagers in the Ejisu-Juabeng district of Ghana. Int J Gynaecol Obstet.

[13] Al-Haddabi R, Al-Bash M, Al-Mabaihsi N, Al-Maqbali N, Al-Dhughaishi T, Abu-Heija A (2014). Obstetric and perinatal outcomes of teenage pregnant women attending a tertiary teaching hospital in Oman. Oman Med J..

[14] Ganchimeg T, Mori R, Ota E, Koyanagi A, Gilmour S, Shibuya K (2013). Maternal and perinatal outcomes among nulliparous adolescents in low- and middle-income countries: a multi-country study. BJOG.

[15] Biney AA (2011). Exploring contraceptive knowledge and use among women experiencing induced abortion in the Greater Accra region, Ghana. Afr J Reprod Health.

[16] Geelhoed D, Nayembil D, Asare K, van Leeuwen JH, van Roosmalen J (2002). Gender and unwanted pregnancy: a community-based study in rural Ghana. J Psychosom Obstet Gynaecol.

[17] Hill ZE, Tawiah-Agyemang C, Kirkwood B (2009). The context of informal abortions in rural Ghana. J Women's Health.

[18] Adjei G, Enuameh Y, Asante KP, Baiden F, Nettey OEA, Abubakari S (2015). Predictors of abortions in rural Ghana: a cross-sectional study. BMC Public Health.

[19] Ilboudo PGC, Somda SMA, Sundby J (2014). Key determinants of induced abortion in women seeking postabortion care in hospital facilities in Ouagadougou, Burkina Faso. Int J Women’s Health.

[20] Ghana Statistical Service (2011). Ghana multiple indicator cluster survey with an enhanced malaria module and biomarker, 2011, final report.

[21] Wallace JM, Luther JS, Milne JS, Aitken RP, Redmer DA, Reynolds LP (2006). Nutritional modulation of adolescent pregnancy outcome – a review. Placenta.

[22] Shah MK, Gee RE, Theall KP (2014). Partner support and impact on birth outcomes among teen pregnancies in the United States. J Pediatr Adolesc Gynecol.

[23] Kouanda S, Coulibaly A, Ouedraogo A, Millogo T, Meda BI, Dumont A (2014). Audit of cesarean delivery in Burkina Faso. Int J Gynaecol Obstet.

[24] Tyrberg RB, Blomberg M, Kjolhede P (2013). Deliveries among teenage women - with emphasis on incidence and mode of delivery: a Swedish national survey from 1973 to 2010. BMC Pregnancy Childbirth.

[25] Dutta I, Joshi P (2013). Maternal and perinatal outcome in teenage vs. Vicenarian primigravidae - a clinical study. J Clin Diagn Res.

[26] Yatich N, Funkhouser E, Ehiri JE, Agbenyega T, Stilles JK, Rayner JC (2010). Malaria, intestinal helminths and other risk factors for stillbirth in Ghana. Infect Dis Obstet Gynecol.

[27] Wiredu EK, Tattey Y (1998). Autopsy studies on still births in Korle Bu teaching hospital. II: causes of death in 93 still births. West Afr J Med.

[28] Iklaki CU, Inaku JU, Ekabua JE, Ekanem EI, Udo AE (2012). Perinatal outcome in unbooked teenage pregnancies in the university of calabar teaching hospital, calabar, Nigeria. ISRN Obstet Gynecol.

[29] Ha YP, Hurt LS, Tawiah-Agyemang C, Kirkwood BR, Edmond KM (2012). Effect of socioeconomic deprivation and health service utilisation on antepartum and intrapartum stillbirth: population cohort study from rural Ghana. PLoS One.

[30] Althabe F, Moore JL, Gibbons L, Berrueta M, Goudar SS, Chomba E, Derman RJ, Patel A, Saleem S, Pasha O, Esamai F, Garces A, Liechty EA, Hambidge K, Krebs NF, Hibberd PL, Goldenberg RL, Koso-Thomas M, Carlo WA, Cafferata ML, Buekens P, McClure EM (2015). Adverse maternal and perinatal outcomes in adolescent pregnancies: the global Network's maternal newborn health registry study. Reprod Health.

[31] Conde-Agudelo A, Belizán JM, Lammers C (2005). Maternal-perinatal morbidity and mortality associated with adolescent pregnancy in Latin America: cross-sectional study. Am J Obstet Gynecol.

[32] Rosenberg M, Pettifor A, Miller WC, Thirumurthy H, Emch M, Afolabi SA (2015). Relationship between school dropout and teen pregnancy among rural south African young women. Int J Epidemiol.

[33] Myer L, Mlobeli R, Cooper D, Smit J, Morroni C (2007). Knowledge and use of emergency contraception among women in the western cape province of South Africa: a cross-sectional study. BMC Womens Health.

[34] Yuan B, Målqvist M, Trygg N, Qian X, Ng N, Thomsen S (2014). What interventions are effective on reducing inequalities in maternal and child health in low- and middle-income settings? A systematic review. BMC Public Health.

